# cGMP and cAMP differentially regulate differentiation and function of brown adipocytes

**DOI:** 10.1186/1471-2210-11-S1-P37

**Published:** 2011-08-01

**Authors:** Katja Jennissen, Bodo Haas, Wolfram S Kunz, Alexander Pfeifer

**Affiliations:** 1 Institute of Pharmacology and Toxicology, Universtiy of Bonn, Bonn, Germany; 2 Devision of Neurochemistry, Department of Epileptology, University of Bonn, Bonn, Germany

## Background

Obesity and associated metabolic disorders result from an imbalance between energy intake and expenditure. White fat is the major site of energy storage in the body. In contrast, brown adipose tissue (BAT), which is highly vascularized and densely packed with mitochondria, is important for energy dissipation by thermogenesis. BAT plays an important role in human neonates in non-shivering thermogenesis as a defense against cold. However, studies using positron emission tomography show that also adult humans have metabolically active BAT. BAT activity is controlled by the sympathetic branch of the autonomic nervous system. Norepinephrine (NE) induces both lipolysis as well as the expression of uncoupling protein-1 (UCP-1) via increasing cellular cAMP levels. UCP-1, which uncouples the proton gradient across the inner mitochondrial membrane from ATP synthesis, is essential for the thermogenic function of BAT. Additionally, NO/cGMP signaling enhances differentiation of brown fat cells through activation of cGMP-dependent protein kinase I [1].

## Methods

Here, we directly compared the effects of cAMP and cGMP on brown fat cell differentiation and their role in brown fat cell function. To investigate the influence on brown adipogenic differentiation, we isolated preadipocytes from interscapular BAT of newborn mice. Brown fat cell differentiation was accomplished using an adipogenic cocktail containing the non-specific PDE inhibitor IBMX. To test whether both nucleotides are required for differentiation, IBMX was replaced by either cGMP, cAMP or both during the phase of adipogenic induction. Additionally, we studied the effects of long-term application of cGMP and cAMP on differentiation as well as acute effects of both cyclic nucleotides on brown fat cell function as assessed by measurement of lipolysis and mitochondrial respiration.

## Results

**Figure 1:**
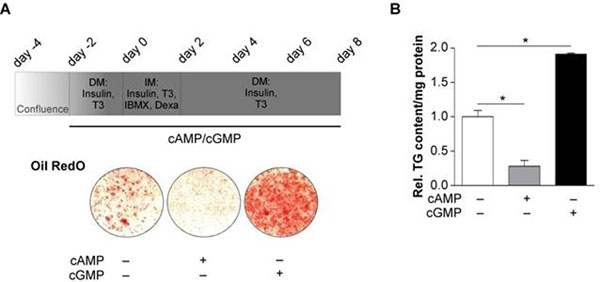
Effects of chronic cAMP and cGMP treatment on brown fat cell differentiation. **A** The protocol for adipogenic differentiation of BAT-derived preadipocytes and the media (DM, differentiation medium; IM, induction medium; Dexa, dexamethasone; IBMX, 3-isobutyl-methyl-xanthine; T3, triiodothyronine) used at the different time points (upper panel). Effects of chronic 8-Br-cAMP (cAMP; 200 µM) and 8-pCPT-cGMP (cGMP; 200 µM) treatment during differentiation of brown preadipocytes on lipid accumulation as assessed by Oil RedO staining (day 7) (lower panel). **B** TG content of differentiated brown adipocytes (day 7) after chronic treatment with cAMP or cGMP during differentiation. Data are represented as means ± SEM; * p < 0.05.

Substitution of IBMX by either cGMP, cAMP or both during the phase of adipogenic induction revealed that only a combination of cGMP and cAMP can accomplish brown adipogenic differentiation as assessed by determination of lipid accumulation and adipogenic marker expression. Nevertheless, chronic application of cAMP during brown fat cell differentiation (Figure 1A) reduced lipid accumulation to 28 ± 9 % of untreated cells (Figure 1B) and decreased the expression of adipogenic markers, namely the peroxisome proliferator-activated receptor γ (PPARγ), which is a master regulator of adipogensis, as well as UCP-1 and the fatty acid-binding protein 4 (aP2). In contrast, application of cGMP resulted in a 1.91 ± 0.02 fold increase of triglyceride content (TG) (Figure 1B) and an elevated expression of adipogenic markers.

Time course experiments revealed that cAMP exerted the most pronounced inhibitory effect on differentiation after the phase of adipogenic induction leading to a decreased expression of PPARγ and lipid accumulation.

Furthermore, we investigated the effects of cGMP and cAMP on brown adipocyte function. Both cGMP and cAMP increased mitochondrial respiration 1.41 ± 0.07 and 1.80 ± 0.20 fold, respectively. Interestingly, cGMP did not stimulate the phosphorylation of hormone-sensitive lipase. Correspondingly, cGMP as well as DEA-NO and the C-type natriuretic peptide did not stimulate lipolysis, whereas cAMP, NE and isoproterenol increased hydrolysis of TGs 1.57 ± 0.19, 2.97 ± 0.32 and 2.61 ± 0.22 fold in murine brown fat cells.

## Conclusion

Taken together, cGMP and cAMP exerted differential effects on brown preadipocyte differentiation and activation of mature brown fat cells. Both cyclic nucleotides were necessary during the induction phase to ensure adipogenic differentiation. Interestingly, chronic application of cAMP resulted in a decrease in differentiation. In contrast, chronic cGMP treatment enhanced adipogenic differentiation. Moreover, cGMP as well as cAMP increased mitochondrial respiration in mature brown adipocytes, but only cAMP was capable of stimulating lipolysis.
